# Emerging Trends in Neoadjuvant Immunotherapy for Hepatocellular Carcinoma: A Focus on Liver Transplant Candidates

**DOI:** 10.1002/cnr2.70244

**Published:** 2025-07-19

**Authors:** Dongdong Yu, Hao Chen, Lidong Wang

**Affiliations:** ^1^ Department of Orthopedic Surgery, First Affiliated Hospital Zhejiang University School of Medicine Hangzhou China; ^2^ Department of Lung Transplantation and Thoracic Surgery, First Affiliated Hospital Zhejiang University School of Medicine Hangzhou China; ^3^ The Department of Hepatobiliary and Pancreatic Surgery, Shulan (Hangzhou) Hospital Affiliated to Zhejiang Shuren University Shulan International Medical College Hangzhou Zhejiang China

## Abstract

**Background:**

Immunotherapy has emerged as a promising neoadjuvant strategy for hepatocellular carcinoma (HCC), with growing evidence supporting its role in tumor downstaging and enabling radical resection. Liver transplantation remains a curative option for HCC, and the integration of neoadjuvant immunotherapy prior to transplantation holds potential to improve staging outcomes and reduce postoperative recurrence. However, this approach necessitates careful evaluation of transplant‐related immunological risks, particularly the risk of allograft rejection.

**Recent Findings:**

Recent clinical trials have provided key data on the efficacy and safety of pre‐transplant immunotherapy. These studies highlight the importance of patient selection and risk management strategies to optimize treatment outcomes. Novel immunotherapeutic approaches are being explored, with a focus on identifying patients most likely to benefit from neoadjuvant therapy prior to liver transplantation.

**Conclusion:**

This review synthesizes emerging trends in the use of neoadjuvant immunotherapy for HCC patients undergoing liver transplantation. While immunotherapy shows promise in enhancing staging success and reducing recurrence, careful consideration of immunological risks and patient‐specific factors is essential. Future research should continue to evaluate the long‐term benefits and safety of this approach, as well as refine strategies for patient selection and risk mitigation. The integration of immunotherapy into pre‐transplant care may represent a transformative advancement in the treatment of HCC, provided these challenges are addressed.

## 1 Introduction

Hepatocellular carcinoma (HCC), the third leading cause of cancer‐related deaths globally, is the fifth most common cancer worldwide [1]. The advent of efficacious systemic therapeutics has markedly enhanced the clinical outcomes for individuals with advanced‐stage HCC [2, 3, 4, 5]. For patients with unresectable HCC who are eligible according to the Milan criteria, orthotopic liver transplantation (LT) has emerged as a highly efficacious standard treatment. The Milan criteria delineate eligibility based on a solitary tumor not exceeding 5 cm in diameter or a maximum of three tumors, each no larger than 3 cm, without vascular invasion or extrahepatic spread [6]. In the United States, organ allocation is reserved for patients who, following tumor downstaging, adhere to the Milan criteria and are subsequently eligible for cadaveric donor allocation. Downstaging/bridging therapies for HCC present an opportunity for LT to patients initially ineligible due to not meeting the Milan criteria [7, 8, 9, 10, 11, 12, 13, 14].

In recent years, immunotherapy, exemplified by immune checkpoint therapy, has emerged as a transformative treatment for HCC [15, 16]. A number of immune checkpoint inhibitors (ICIs), including antibodies targeting programmed death protein‐1 (PD‐1) and its ligand (PD‐L1), as well as antibodies against cytotoxic T‐lymphocyte‐associated protein 4 (CTLA‐4), have received approval for the treatment of HCC [15, 17]. Moreover, in addition to immune checkpoints, immunotherapeutic strategies such as tumor vaccines, lysosomal virus immunotherapy, and Adoptive Cell Transfer Therapy are also being investigated for the treatment of HCC [18, 19]. Immune checkpoints serve as a regulatory mechanism within the immune system, preventing excessive activation and potential damage to host tissues under normal conditions. However, there are instances where tumor cells exploit these checkpoints to circumvent the immune system [20]. The blockade of inhibitory signals enables the restoration of T‐cell activity, thereby facilitating the recognition and attack of tumor cells. Given the favorable efficacy in advanced HCC, immune checkpoint therapy has also been explored as a preoperative adjuvant therapy for HCC. Numerous phase I/II clinical trials have explored the synergistic effects of immunotherapy in conjunction with surgical or local treatments for early‐to intermediate‐stage HCC, reporting promising outcomes [12, 21, 22]. However, the role of immunotherapy as a preoperative adjuvant in the context of LT for HCC continues to be a contentious issue. Although preliminary evidence suggests that pre‐transplant immunotherapy may augment the likelihood of successful downstaging and enhance tumor control, it also poses significant immunological challenges post‐transplantation. Therefore, the strategic deployment of neoadjuvant immunotherapy, with meticulous attention to critical variables such as the timing of administration, dosage, and therapeutic combinations, is imperative for achieving the most favorable therapeutic outcomes.

## 2 Potential Application of Neoadjuvant Immunotherapy in LT

While LT presents a potentially curative treatment for HCC, a significant dropout rate of approximately 30% from the waiting list is observed [23]. This high attrition is attributed to various factors, including an average waiting period of 6 months, extended delays due to organ scarcity, and disease progression [24, 25]. These findings underscore the imperative for the implementation of pre‐transplantation therapies for HCC. Neoadjuvant therapy encompasses downstaging to alleviate tumor burden and meet transplant criteria (Milan criteria), as well as bridging therapy to manage tumors during the organ allocation process. Locoregional interventions, such as transcatheter arterial chemoembolization (TACE) and radiofrequency ablation, currently constitute the primary modalities for both downstaging and bridging therapies [26, 27]. However, the emerging evidence supporting the efficacy of immunotherapy as a systemic treatment has garnered increasing interest in its potential role as an alternative or adjunctive strategy in the management of HCC [8, 11].

Furthermore, residual micrometastases contribute to disease recurrence in approximately 10%–20% of patients following LT [28]. Neoadjuvant immunotherapy has the potential to effectively activate anti‐tumor CD8+ T cells prior to the establishment of widespread metastasis, when functional lymphoid structures are still intact. This intervention may enhance the immune system's capacity to detect and eliminate micrometastatic foci, thereby reducing the risk of post‐transplantation recurrence [29].

## 3 Immunotherapy Combinations Significantly Improve Survival in Unresectable HCC

Recent Phase III trials have demonstrated the significant efficacy of immune‐based combination therapies in unresectable HCC. The EMERALD‐1 trial (*n* = 616) showed that TACE combined with durvalumab and bevacizumab significantly prolonged median progression‐free survival (PFS) (6.8 months vs. TACE alone, Hazard Ratio (HR) = 0.77, *p* = 0.032) and improved the objective response rate (ORR) to 44% [30]. The LEAP012 trial (*n* = 480) confirmed that TACE combined with lenvatinib and pembrolizumab achieved a median PFS of 14.6 months (vs. 10 months with TACE alone, HR = 0.66, *p* = 0.0002) and an ORR of 47% [31]. Long‐term follow‐up of the HIMALAYA trial (*n* = 1171) at 49.1 months demonstrated that the STRIDE regimen (single‐dose tremelimumab combined with durvalumab) significantly improved overall survival (OS) compared to sorafenib (HR = 0.78), with a 36‐month survival rate of 30.7%, and 57.3% of long‐term survivors requiring no further therapy [32]. The IMbrave150 trial (*n* = 501) showed that atezolizumab combined with bevacizumab significantly reduced the risk of death by 42% compared to sorafenib (HR = 0.58, *p* < 0.001), with a median OS not yet reached (vs. 13.2 months) [33]. The CARES‐310 trial (*n* = 543) further validated the survival benefit of camrelizumab combined with apatinib, achieving a median OS of 22.1 months (vs. 1.52 months with sorafenib, HR = 0.62, *p* < 0.0001) [34]. These findings provide a robust evidence base for the application of immune combination therapies in neoadjuvant settings for liver cancer, particularly highlighting synergy effects of PD‐1/PD‐L1 inhibitors combined with anti‐angiogenic agents or CTLA‐4 inhibitors.

## 4 Immunotherapy and Surgical Resection Therapy

Immunotherapy has emerged as a potent downstaging strategy in HCC, as evidenced by a wealth of clinical trial data. In the context of unresectable HCC, the integration of ICIs into treatment regimens has consistently demonstrated response rates exceeding 30% across multiple studies [22, 34, 35, 36]. The efficacy of neoadjuvant immunotherapy in HCC has been substantiated by three pivotal trials. In a single‐arm, phase 1b trial, the utility of neoadjuvant cabozantinib combined with nivolumab for the downstaging of unresectable HCC was evaluated. Among the 15 enrolled patients, an impressive 80% underwent successful margin‐negative resection, with 42% (5 out of 12) exhibiting a profound pathological response, characterized by tumor necrosis of at least 90% [37]. In a parallel trial, patients with resectable HCC (stages Ib, II, and IIIb) were administered two cycles of neoadjuvant cemiplimab monotherapy prior to surgery, followed by surgical resection and an additional eight cycles postoperatively. Among the 20 patients who had a tumor resection, 20% experienced substantial tumor necrosis, 15% achieved a partial response, and the remainder maintained stability [22]. Another study investigated the administration of nivolumab, with or without ipilimumab, to patients 6 weeks preoperatively and continued for up to 2 years postoperatively. Significantly, 33% of the 9 patients who received nivolumab monotherapy exhibited severe tumor necrosis, while 27% of the 11 patients treated with the combination of nivolumab and ipilimumab showed the same. In addition, the estimated median PFS was 9.4 months (95% confidence interval [CI] 1.47) for nivolumab and 19.53 months (2.33) for nivolumab and ipilimumab (HR 0.99, 95% CI 0.31–2.54) [38]. In response to these clinical findings, it is important to emphasize the limitations of the statistical significance of neoadjuvant therapy, despite the fact that it induced significant pathological remissions in some patients. Considering that small sample sizes lead to wide confidence intervals and insufficient statistical power, future prospective studies with larger samples are needed to confirm their statistical significance. Recently, the VITALITY study included 177 prospective cases of patients treated with ICIs with the aim of undergoing LT. Despite a 50% dropout rate, the 3‐year intention‐to‐treat survival rate was 71.1%. Notably, patients within the Milan criteria demonstrated a survival rate of 73.5%, compared to 69.7% in patients exceeding the Milan criteria (*p* = 0.329) [39]. The results of this first multicenter evaluation, which showed promising intention‐to‐treat survival rates, provide reassuring evidence of the clinical significance of the use of ICIs prior to transplantation.

The potential of local treatment combined with immunotherapy to enhance the respectability of HCC is exemplified by the START‐FIT trial, which was a phase 2, single‐arm study. This trial assessed the efficacy of a sequential treatment approach involving TACE and stereotactic body radiotherapy, culminating in avelumab administration—an anti‐PD‐L1 therapy—for locally advanced, unresectable HCC. The study enrolled 33 patients, 64% of whom presented with macrovascular invasion, with a median follow‐up time of 17.2 months. Encouragingly, 55% of the cohort were deemed suitable for radical treatment after the intervention [40].

While these studies do not specifically target liver transplant candidates, the patient populations studied, characterized by intermediate‐stage HCC, bear resemblance to those undergoing LT. Consequently, it is reasonable to infer that immunotherapy could potentially expand the pool of LT candidates or mitigate the risk of waitlist dropout by facilitating downstaging. These preliminary findings underscore the burgeoning role of immunotherapy as an adjunctive preoperative treatment modality.

In addition, preoperative neoadjuvant immunotherapy may similarly reduce postoperative recurrence. In a phase 2 trial evaluating the role of the PD‐1 inhibitor sintilimab in the preoperative neoadjuvant treatment of HCC with microvascular infiltration (MVI), 99 eligible patients received neoadjuvant treatment with sintilimab. Sintilimab significantly prolonged RFS compared with active surveillance (median RFS, 27.7 versus 15.5 months; Risk ratio 0.534, 95% CI 0.360–0.792; *p* = 0.002). The potential effectiveness of ICIs as adjuvant therapy for high‐risk patients is supported by these findings [41].

## 5 Safety of Immunotherapy

**FIGURE 1:**
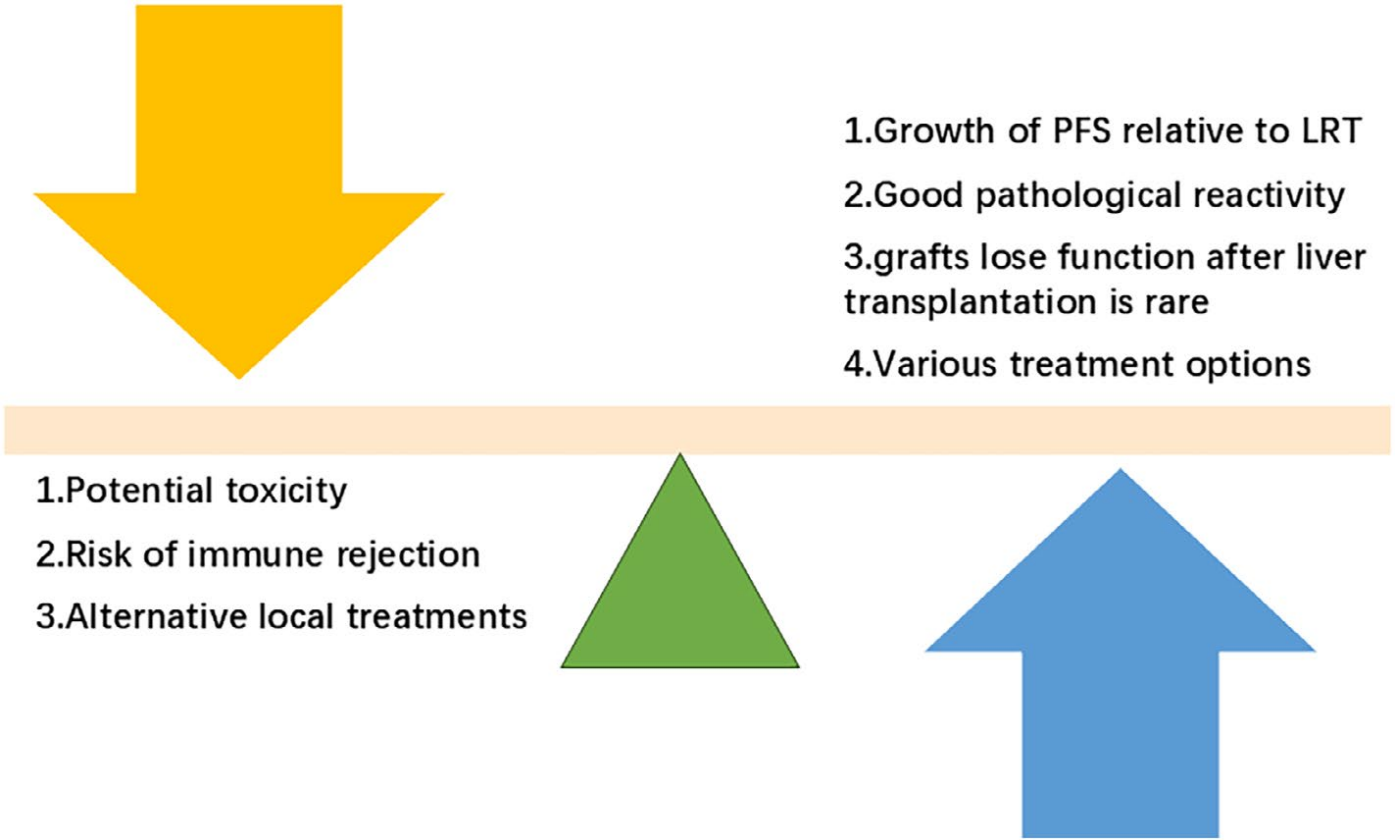
Risks versus benefits of Neoadjuvant Immunotherapy for HCC in the liver transplant population. HCC: hepatocellular carcinoma; LRT: locoregional therapies; PFS: progression‐free survival.

Several retrospective studies have confirmed the safety of immunotherapy prior to liver LT [42, 43, 44, 45, 46, 47, 48, 49]. Review by Luca Marzi summarized most of the current clinical studies on rejection after LT with preoperative immunotherapy [50]. Tabrizian et al. reported nine patients who received nivolumab monotherapy followed by LT, of whom only one developed mild acute rejection [42]. Tabrizian et al. also outlined in their review the results of a multicenter study evaluating 80 patients with HCC treated with ICIs for LT. At the time of the abstract's publication, 30 patients had undergone LT, with five patients (16.7%) experiencing rejection and only one case of graft loss (3.3%) [51]. Wang et al. described 16 patients treated with anti‐PD‐1 monotherapy; although acute rejection (defined by elevated liver function tests rather than histology) occurred in nine patients, no cases of graft loss were reported [43]. Additionally, several larger studies have confirmed the safety of preoperative neoadjuvant immunotherapy. In a meta‐analysis of 91 cases in which ICIs were used prior to LT, the rate of allograft rejection was 26.4%, with 83% of patients recovering with medication. No difference in survival after LT was observed when comparing patients with and without rejection (HR, 2.16 [95% CI, 0.58 to 8.1]) [29]. Another multicenter retrospective cohort study reported 83 patients from 11 centers who underwent preoperative LT with ICIs. After a mean follow‐up of 8.1 months (interquartile range, 3.3–14.6), a rejection rate of 27.7% was observed [52]. Both studies found that a longer washout period was associated with a lower risk of rejection, while no significant association was observed between rejection and immunosuppressive regimens. More recently, the VITYITAL study included 177 cases of prospectively treated LT with ICIs. Reassuringly, the rejection rate in transplant patients (*n* = 43) was 16.3%, with no deaths attributed to rejection [39]. Considering that the risk of rejection after LT with or without ICIs ranges from 15.6% to 26.9% [53], and is as high as 64% in biopsy cohorts [54], such rejection rates are deemed acceptable. Current data suggest that the safety of immunotherapy as a neoadjuvant treatment preceding LT is manageable. However, its efficacy and safety profile must be substantiated through rigorous, large‐scale clinical trials to establish its definitive role in pretransplantation therapeutic regimens. The balance between the safety and efficacy of immunotherapy must be carefully considered, as illustrated in Figure 1.

## 6 How to Select Suitable Liver Cancer Liver Transplant Patients for Neoadjuvant Immunotherapy

The judicious application of neoadjuvant immunotherapy necessitates a thorough evaluation of both its efficacy and safety. Clinical data from previous studies underscore the pivotal role of patient selection in optimizing the outcomes of immunotherapy [12]. A meta‐analysis, comprehensive in scope, of phase III randomized controlled trials that took place from 2002 to 2020, which examined systemic therapies for HCC across various stages, indicated that immunotherapy might exhibit enhanced efficacy in virus‐associated HCC cases [55]. In a phase II, single‐arm, prospective study with pembrolizumab, tumor biopsy samples obtained prior to treatment from a cohort of 60 HCC patients who had previously failed sorafenib therapy were subjected to extensive molecular characterization. Notably, patients with cytotoxic T‐cell infiltration within the tumor microenvironment, as well as those exhibiting elevated levels of active circulating CD8+ T cells coupled with downregulation of neutrophil‐associated markers, were found to derive significant clinical benefit from pembrolizumab treatment [56]. Furthermore, ongoing research is delving into the identification of novel predictive biomarkers. This includes advanced techniques such as genomic sequencing, single‐cell RNA sequencing, immune cell phenotyping, radiomics, and microbiome analysis [57]. Genetic traits such as tumor mutational load (TMB), microsatellite instability, and mismatch repair defects have been shown to correlate with response to ICIs [58, 59, 60]. For example, in HCC, patients with tumors with higher TMB respond better to ICI and have longer survival [58]. In addition, immune cell infiltration in the tumor microenvironment can likewise serve as an important predictor of ICIs efficacy [61], such as the level of CD8+ T cells and PD‐L1 expression. Detailed mapping of subclassification characteristics, cell type communities present, and spatial relationships of tumor biopsy samples extracted by transcriptomes, single‐cell transcriptomics, and spatial transcriptomics offer the possibility of developing new biomarkers [62, 63, 64]. To better guide clinical decision‐making, the application of biomarkers needs to be more systematic. For example, the CRAFITY score, which combines alpha‐fetoprotein (AFP) and C‐reactive protein levels, is effective in predicting the prognosis of HCC patients receiving immunotherapy [65, 66, 67]. This comprehensive scoring method can be an important tool for patient selection. Tumor dynamics are monitored in real time through liquid biopsy techniques such as circulating tumor DNA and circulating tumor cells and other non‐invasive methods [56, 68, 69]. In addition, comprehensive models capable of predicting treatment response can be developed through big data analysis and machine learning algorithms [70]. Greten et al. summarized the biomarkers relevant to the treatment of ICIs in HCC [57]. A significant advantage of neoadjuvant therapies is the accessibility to surgically resected tumor tissues from the majority of patients, facilitating in‐depth biomarker analysis. Consequently, the potential of biomarker‐guided immunotherapy for LT in HCC can be rigorously explored as pertinent clinical trials are underway.

The safety profile of neoadjuvant immunotherapy in the context of LT for HCC is primarily contingent upon the management of immune‐related adverse events (irAEs) and the risk of potential graft rejection. ICIs may induce multi‐systemic toxicity, with common manifestations in the skin, heart, gastrointestinal tract, lungs, liver, and endocrine organs [71, 72]. Notably, approximately 10%–20% of patients receiving PD‐1/PD‐L1 antibody therapy and up to 25% of those treated with CTLA‐4 antibodies encounter severe irAEs [73, 74, 75]. Fatigue, cutaneous reactions, and hepatic injury are among the most frequently observed irAEs. Moreover, when PD‐1/PD‐L1 antibodies are used in combination with CTLA‐4 antibodies, synergistic immunotoxicity emerges in nearly 50% of HCC patients [76]. In a phase II trial evaluating the combination therapy of nivolumab and ipilimumab, the necessity for corticosteroid intervention was highlighted, often in response to severe irAEs [76]. Furthermore, when ICIs are co‐administered with anti‐angiogenic agents, such as tyrosine kinase inhibitors, the potential for cumulative and non‐overlapping toxicities exists, with the rate of serious adverse events reaching as high as 67% [77].

The impact of irAEs is particularly pronounced in patients with pre‐existing autoimmune diseases or those undergoing transplantation [78]. Therefore, the management of irAEs requires accurate diagnosis and rigorous grading according to the Common Terminology Criteria for Adverse Events developed by the National Cancer Institute [79]. The grading of irAEs depends on the treatment regimen used. IrAEs are generally dose‐dependent when using regimens containing anti‐CTLA‐4 antibodies (30%–55% with ipilimumab + nivolumab combinations), but they are not dose‐dependent with anti‐PD‐1/PD‐L1 monotherapy (10%–15% incidence) [80]. IrAEs typically occur within the first 3 months of treatment but may arise at any time. For acute irAEs, although not supported by large‐scale randomized controlled trials, management involves discontinuing ICIs and administering high‐dose glucocorticoids (or other immunosuppressive agents for steroid‐refractory cases) [79, 81]. While this treatment does not appear to interfere with antitumor responses, re‐initiating steroid administration during treatment may lead to adverse outcomes [82, 83, 84]. Although acute irAEs have garnered significant attention due to their clinical urgency and need for prompt intervention, chronic irAEs may also play a critical role in the pre‐transplantation immunotherapy setting, as they may persist or emerge post‐transplantation. Retrospective data published in 2021 showed that chronic irAEs (defined as lasting > 12 weeks after immunotherapy discontinuation) occurred in 43.2% of patients [85]. Additionally, delayed/late‐onset irAEs (occurring more than 3 months after cessation of immunotherapy) warrant attention in monitoring for potential post‐transplantation irAEs. Multidisciplinary care is indispensable for patients with HCC treated with ICIs due to the complexities of managing chronic liver disease alongside liver‐specific and systemic symptoms [86].

Postoperative rejection in liver transplant patients who received ICIs preoperatively is a significant concern. A retrospective study demonstrated that liver transplant rejection occurred in 37.5% of cases, and 75% of patients ultimately developed organ failure [87]. Preliminary data suggest that the temporal relationship between the last immunotherapy infusion and transplantation significantly impacts transplantation outcomes, and the risk of immunotherapy‐related graft rejection may be mitigated by optimizing this interval. In three separate case series, the incidence of rejection was significantly higher (75%) when the interval between nivolumab administration and transplantation was 16 days, compared to 12.5% and 0% when the interval was 28 days and 90 days or more, respectively [44, 88]. Based on preclinical evidence and the 15‐ to 27‐day half‐life of ICIs (Table 1), a “washout period” of 90 days after ICI treatment appears to be a prudent strategy to minimize the risk of severe rejection after transplantation. It is important to recognize, however, that the immunomodulatory effects of ICIs may persist beyond their half‐life, and their therapeutic effects and potential for irAEs can last for 6–12 months after discontinuation. Although absolute safety cannot be guaranteed during the washout period, the efficacy of this approach in reducing rejection risk remains under active investigation [89, 90, 91]. The applicability of these pharmacokinetic insights to dual‐drug regimens, such as atezolizumab plus bevacizumab, remains to be determined. Given that patients with autoimmune diseases have historically been excluded from clinical trials, caution is warranted when administering neoadjuvant immunotherapies to this population, with careful consideration of the risks of disease recurrence and rejection following prolonged chemotherapy. Additionally, the management of acute rejection after LT typically follows established guidelines [47, 70], with methylprednisolone as first‐line therapy demonstrating a high success rate in reversing rejection. In steroid‐resistant cases, anti‐thymocyte globulin (ATG) has shown efficacy, and plasma exchange may improve outcomes by removing ICIs from the system. Adjusting baseline immunosuppression improves outcomes; however, patient responses vary widely, and some patients may ultimately require retransplantation [43, 92, 93].

**TABLE 1:** Half‐life period of immunotherapy used for HCC [45].

Immunotherapy agent	Mechanism of action	Estimated half‐life (days)
Nivolumab	PD‐1 inhibitor	26.7
Pembrolizumab	PD‐1 inhibitor	23
Ipilimumab	CTLA‐4 inhibitor	15.4
Atezolizumab	PD‐L1 inhibitor	27
Durvalumab	PD‐L1 inhibitor	18

Abbreviations: CTLA‐4, cytotoxic T‐lymphocyte–associated protein 4; PD‐1, programmed cell death‐1; PDL‐1, programmed cell death ligand‐1.

Immunosuppression regimens for patients who received immunotherapy prior to LT typically involve drug combinations designed to minimize rejection risk. Initial induction therapy usually consists of methylprednisolone, followed by maintenance regimens involving mycophenolate mofetil and a calcineurin inhibitor or, less commonly, a mammalian target of rapamycin inhibitor. Prednisone is typically tapered over several weeks. Some regimens also include additional agents such as basiliximab or ATG during induction [46, 48].

The inclusion criteria from several randomized controlled trials, including ImmunoXXL [12], PLENTY, and ESR‐20‐21010, which meet the University of California, San Francisco criteria [94], can serve as a reference for patient screening for neoadjuvant immunotherapy in LT. As numerous studies are currently exploring the efficacy and safety of neoadjuvant immunotherapy prior to LT, there is an urgent need to discern which HCC patients should be categorized as “high risk.” The use of pre‐LT immunotherapy will be deemed ‘high risk’ unless robust data emerge demonstrating that HCC patients considered for LT achieve superior clinical outcomes with neoadjuvant immunotherapy compared to locoregional therapy and surveillance.

## 7 New Immunotherapy Protocols in LT for HCC

### 7.1 Cancer Vaccine

Historically, cancer prophylactic vaccines, particularly those targeting virus‐associated malignancies, have been instrumental in reducing the incidence of certain virus‐driven cancers. Notably, vaccines against human papillomavirus and hepatitis B virus (HBV) have contributed to a substantial reduction in the incidence of HCC associated with HBV [95]. In recent years, the therapeutic landscape has expanded to include personalized cancer vaccines (PTCV) for the treatment of HCC. A phase 1/2 clinical trial with a single‐arm, open‐label design assessed the safety and effectiveness of a DNA plasmid vaccine, PTCV (GNOS‐PV02), which encodes up to 40 neoantigens. The vaccine was administered alongside interleukin‐12 and pembrolizumab in advanced HCC patients who had already received treatment with a multi‐tyrosine kinase inhibitor. The trial involved 36 patients and reported no dose‐limiting side effects or high‐grade adverse reactions associated with therapy. The objective remission rate, as per the modified intention‐to‐treat analysis and Criterion 1.1 of the Solid Tumor Efficacy Assessment, was 30.6% (11 of 36 patients), with complete remission achieved in 8.3% (3 of 36 patients). These results support the mechanism of PTCV, which is based on the induction of anti‐tumor T‐cell responses, and propose that the co‐administration of PTCV and pembrolizumab gains significant therapeutic results in advanced HCC [19]. Furthermore, another study designed and administered a PTCV to 10 HCC patients pinpointed with a high susceptibility to postoperative recurrence. During the neoantigen vaccination phase, there were no major side effects noticed among the patients. Clinical recurrence was confirmed by imaging in 8 of the 10 patients, while the remaining 2 patients showed no signs of recurrence before the cut‐off date. The median recurrence‐free survival (RFS) for the 10 patients was 7.4 months from the first neoantigen vaccination. Among the seven patients who completed all scheduled neoantigen vaccinations, five demonstrated T‐cell responses induced by the neoantigens and had a significantly longer RFS after radical surgery compared to the other patients who did not exhibit reactive neoantigens or who received only primary immunization, with propensity scores matched to those of the control patients (*p* < 0.05) [96].

### 7.2 Natural Immune Cell Transfer Therapy

Natural immune cell transfer therapy has emerged as a promising treatment modality for HCC [97, 98, 99]. In a pivotal trial, 150 patients who had radical resection for HCC were assigned at random to either receive adjuvant immunotherapy or without supplementary treatment. The adjuvant therapy group was treated with five infusions of autologous lymphocytes activated ex vivo with recombinant interleukin‐2 and CD3 antibodies within 6 months post‐surgery. This adjuvant approach successfully delayed the median time to first relapse (2.8 vs. 1.6 years; *p* < 0.01) and improved 5‐year relapse‐free survival (RFS; 37% vs. 22%; *p* < 0.01), although it did not significantly impact OS [100]. A meta‐analysis encompassing six randomized controlled trials assessed the safety and efficacy of cytokine‐induced killer (CIK) cells. The analysis revealed that CIK cells not only enhanced disease‐free survival in the first 3 years but also improved OS in the first 3 years. Moreover, CIK cell therapy was determined to be a secure approach [101]. An open, randomized, controlled trial investigated the efficacy of autologous invariant natural killer T (iNKT) cells in advanced HCC patients following the failure of TACE. Patients were assigned to receive TACE with fortnightly iNKT cell infusions or TACE alone. The iNKT cell group exhibited a significantly higher median PFS (5.7 months) compared to the TACE‐only group (2.7 months; *p* < 0.001). ORR and disease control rate (DCR) were also superior in the iNKT cell group (52% and 85%, respectively) versus the TACE group (11% and 33%, respectively). Complete remission was achieved in five iNKT cell recipients and one TACE patient. The average duration until a decline in life quality was greater for the iNKT cell group, with a median of 9.2 months, compared to the TACE group, which had a median of 3.0 months. Serious side effects (above Grade 3) were rare, occurring in only one iNKT cell recipient (4%) and 5 TACE patients (19%), with all other adverse events being grade 1–2. iNKT cell infusion significantly improved PFS, ORR, DCR, and quality of life with manageable toxicity [102].

### 7.3 T‐Cell Receptor Engineered T Cell Therapy (TCR‐T)

TCR‐T cells are a new type of immunotherapy that uses natural T‐cell receptors (TCRs) that can target tumor antigens to modify the patient's own T‐cells so that they can specifically recognize and attack tumor cells [103, 104, 105]. Targeting AFP‐specific TCR‐T cell therapy and Hepatitis C virus‐specific TCR‐T cells have shown significant killing ability against HCC both in vitro and in vivo [106, 107, 108, 109]. HBV mutant antigen‐associated TCR‐T cell therapy is the most widely used TCR‐T therapy against HCC. In 2011, Gehring et al. successfully generated TCR‐T cells specific to HBV epitope antigens using peripheral blood mononuclear cells from patients with chronic HBV infection and HBV‐associated HCC. These HBV‐specific TCR‐T cells not only targeted HBV‐associated HCC cells effectively but also had the potential to restore virus‐specific T‐cell immunity in patients with chronic HBV infection [110]. Building on this foundation, Qi Liu et al. subsequently developed hepatitis B surface antigen (HBsAg)‐specific artificial intelligence (Ai)‐TCR‐T cells. In a xenograft mouse model of HCC, the infusion of these cells led to the complete elimination of tumors without any observed relapse. The study also revealed a positive correlation between the increased tumor infiltration of CD8+ Ai‐TCR‐T cells and tumor regression. Extensive in vitro and in vivo studies confirmed that these HBsAg‐specific Ai‐TCR‐T cells maintained a safety profile comparable to that of wild‐type cells while exhibiting a significantly enhanced therapeutic efficacy [111]. In the clinical setting of LT, Waseem Qasim et al. addressed chemotherapy‐resistant extrahepatic metastases post‐transplant by employing T‐cell therapy with T cells expressing HBsAg‐specific TCRs. In a separate approach, Morteza Hafezi et al. employed electroporation to introduce mRNA encoding specific TCRs, along with mutated forms of calmodulin B and inosine‐5′‐monophosphate dehydrogenase, into T cells. This strategy aimed to engineer T cells that are specific to HBV or Epstein–Barr virus and resistant to immunosuppressive drugs, thereby providing a novel means to prevent HCC recurrence following LT [112, 113].

### 7.4 Chimeric Antigen Receptor (CAR)‐T/CAR‐Natural Killer (NK)

Genetically engineered CARs can recognize antigens naturally expressed on the surface of cancer cells without the need for TCRs to specifically recognize major histocompatibility complex that present cancerous antigens, and can directly activate T cells to kill cancer cells. This technology is clinically applied to modify T cells/NK cells to develop tumor‐specific immune cells [114, 115]. CAR‐T cells selectively targeting Glypican‐3 (GPC3) are the most studied treatment for HCC. Multiple clinical results confirm the use of GPC‐3 CAR‐T cells to de‐suppress HCC tumors [116, 117]. In addition, new antigenic targets have been used for HCC CAR‐T therapy. A phase II study explored the use of CD133‐directed CAR T cells in adult HCC. Twenty‐one patients with advanced HCC received CART cell infusions. The median OS was 12 months (95% CI, 9.3–15.3 months) and the median PFS was 6.8 months (95% CI, 4.3–8.4 months). The majority of patients were controlled, and only six had progression [118]. Min Yu et al. successfully developed CAR‐modified NK cells targeting GPC3. Their study demonstrated that these GPC3‐targeted CAR‐NK cells significantly reduced the number of tumor infiltrations in GPC3‐positive HCC xenografts and were highly effective in tumor cell lysis [119].

Given the current limitations of immune checkpoint therapy in the context of HCC LT, the development of new immunotherapies is of paramount importance for this patient population. The efficacy of cellular immunotherapy can be improved by a variety of strategies such as genetically engineering immune cells to express/silence chemokines, combining checkpoint inhibitors, genetically engineering high expression of stimulatory factors, and inhibiting the expression of inhibitory signals [120, 121]. The potential occurrence of acute rejection can even be addressed by performing Clustered Regularly Interspaced Short Palindromic Repeats‐system‐mediated genetic engineering modifications to prevent the expression of endogenous human leukocyte antigen (HLA) class I complexes and overexpressing single‐chained multiprotein complexes consisting of β‐2 microglobulin linked to HLA‐E to address allogeneic rejection [122, 123].

Although recent clinical trials exploring neoadjuvant immunotherapy prior to LT for HCC have demonstrated encouraging preliminary outcomes, the statistical validity of these findings is constrained by the single‐arm design and sample limited size of these studies (Table 2). The absence of control groups and restricted cohort sizes introduces ambiguity in attributing observed outcomes solely to the therapeutic intervention, thereby heightening the likelihood of Type I and Type II errors and undermining the reliability of the statistical analysis. To robustly evaluate the therapeutic potential and statistical validity of neoadjuvant immunotherapy in this context, future trials should incorporate larger, well‐powered cohorts alongside appropriately designed control groups. Such methodological refinements will be critical to establishing conclusive evidence and guiding clinical decision‐making in the evolving landscape of HCC management.

**TABLE 2:** Current clinical trials of neoadjuvant immunotherapy in LT for HCC.

Cellular products	Sample size	Stage	Recipients	Status	Year (start‐estimated completion)	Trial ID
Anti‐PD‐1 inhibitor	59	Phase 2	HCC beyond Milan Criteria	Recruiting	2023–2028	NCT05475613
Camrelizumab (SHR‐1210)	120	Phase 1/2	HCC before LT	Unknown	2019–2021	NCT04035876
TCR‐T	13	Phase 1	HBV‐related HCC Post LT	Completed	2018–2021	NCT02686372
Oral PD‐L1 inhibitor (INCB099280)	NA	Phase 1	HCC that is within criteria for LT	Withdrawn	2024–2028	NCT06337162
Atezolizumab (with Bevacizumab)	24	Phase 4	HCC beyond Milan Criteria	Recruiting	2023–2027	NCT05185505
Atezolizmab (with Bevacizumab)	12	Observational	HCC beyond current transplant criteria	Recruiting	2022–2024	NCT05879328
Durvalumab and Tremelimumab	30	Phase 2	HCC in Patients Listed for a Liver Transplant	Recruiting	2021–2030	NCT05027425
ICIs	160	Observational	LT for HCC	Recruiting	2023–2023	NCT05913583

Abbreviations: HBV: hepatitis B virus; HCC: hepatocellular carcinoma; ICIs: immune checkpoint inhibitors; LT: liver transplantation; PD‐1: programmed death‐1; PD‐L1: programmed cell death 1 ligand‐1; TCR‐T: T cell receptor‐gene engineered T cells.

## 8 Conclusion

Neoadjuvant immunotherapy has demonstrated revolutionary potential in the field of HCC LT, and its core value lies in enhancing the success rate of tumor downstaging, expanding the population eligible for radical surgery, and significantly reducing the risk of postoperative recurrence, which provides a new direction to improve long‐term patient survival. However, the possible risk of rejection and the effectiveness of immunotherapy have limited its clinical application. Its full clinical potential can currently only be realized through careful patient selection, rigorous safety assessment, and a deeper understanding of predictive biomarkers. These factors are critical for optimizing treatment strategies and minimizing adverse effects in this patient population. Looking forward, the field awaits the results of ongoing prospective clinical trials that are expected to provide further insights into the efficacy and safety of preoperative immunotherapy in HCC patients undergoing LT. In addition, the continued development of new biomarkers and therapeutic strategies is expected to improve patient outcomes and address current limitations. Future studies should focus on refining predictive models, identifying subgroups of patients most likely to benefit, and exploring combination therapies to overcome drug resistance. Ultimately, these efforts may pave the way for a more precise and effective preoperative neoadjuvant immunotherapeutic approach to LT, changing the landscape of HCC treatment in the context of LT.

## Author Contributions

**Lidong Wang:** conceptualization, literature synthesis, writing – original draft. **Hao Chen:** methodology design, critical analysis. **Dongdong Yu:** supervision, writing – review and editing, conceptualization, literature synthesis, writing – original draft.

## Conflicts of Interest

The authors declare no conflicts of interest.

## Data Availability

No datasets were generated or analyzed during the current study.
